# HaRD: a heterogeneity-aware replica deletion for HDFS

**DOI:** 10.1186/s40537-019-0256-6

**Published:** 2019-10-21

**Authors:** Hilmi Egemen Ciritoglu, John Murphy, Christina Thorpe

**Affiliations:** 1 Performance Engineering Laboratory, School of Computer Science, University College Dublin, Dublin, Ireland; 2 Technological University Dublin, Dublin, Ireland

**Keywords:** Hadoop distributed file system (HDFS), Replication factor, Replica management framework, Software performance

## Abstract

The Hadoop distributed file system (HDFS) is responsible for storing very large data-sets reliably on clusters of commodity machines. The HDFS takes advantage of replication to serve data requested by clients with high throughput. Data replication is a trade-off between better data availability and higher disk usage. Recent studies propose different data replication management frameworks that alter the replication factor of files dynamically in response to the popularity of the data, keeping more replicas for in-demand data to enhance the overall performance of the system. When data gets less popular, these schemes reduce the replication factor, which changes the data distribution and leads to unbalanced data distribution. Such an unbalanced data distribution causes hot spots, low data locality and excessive network usage in the cluster. In this work, we first confirm that reducing the replication factor causes unbalanced data distribution when using Hadoop’s default replica deletion scheme. Then, we show that even keeping a balanced data distribution using WBRD (data-distribution-aware replica deletion scheme) that we proposed in previous work performs sub-optimally on heterogeneous clusters. In order to overcome this issue, we propose a heterogeneity-aware replica deletion scheme (HaRD). HaRD considers the nodes’ processing capabilities when deleting replicas; hence it stores more replicas on the more powerful nodes. We implemented HaRD on top of HDFS and conducted a performance evaluation on a 23-node dedicated heterogeneous cluster. Our results show that HaRD reduced execution time by up to 60%, and 17% when compared to Hadoop and WBRD, respectively.

## Introduction

In recent years, the number of data sources is increasing exponentially (e.g., IoT devices and social media applications), and data is incessantly produced every second. Thus, the volume of data is growing rapidly. Moreover, processing enlarging data-sets has paramount importance for businesses as it helps to determine mission-critical objectives and discover opportunities. Consequently, processing large data-sets in order to extract meaningful information has become vital for business success and has created the demand for large-scale distributed data-intensive systems [1–3].

Apache Hadoop [4] is the de facto framework for large-scale distributed data-intensive computing that employs the MapReduce paradigm [5]. The Hadoop project is composed of 4 main components: (i) Hadoop distributed file system (HDFS) [6], (ii) resource management framework (YARN) [7], (iii) execution engine, and (iv) Hadoop common. The component-based approach of Hadoop helps to use the infrastructure more effectively by making use of more sophisticated components, e.g., Apache Spark [8] can be used instead of the MapReduce engine as it allows in-memory processing of the data. HDFS proved to be a highly scalable, robust distributed storage system in the big data ecosystem. Therefore, companies trust in HDFS to store their petabytes of data reliably on distributed nodes. HDFS not only serves as a reliable storage system but also provides high throughput for thousands of clients’ concurrent queries. Data stored in HDFS can be retrieved by simple MapReduce jobs or complex graph processing jobs. Thus, the performance of HDFS is a critical matter for the whole big data ecosystem that stands on HDFS.

The key idea behind the robustness and efficiency of HDFS is the distributed placement of replicated data. Any file stored on HDFS is divided into fixed-size blocks (chunks). Each block is stored by replicating three times (by default). Moreover, each replica is distributed among different nodes in the cluster. This strategy advances system performance through effective load-balancing and provides fault-tolerance [9, 10]. Hence, different replica management frameworks have been proposed in the literature to improve the system performance by adapting the replication factor either proactively [11], or dynamically [12–14] depending on the popularity of data. Existing replica management frameworks increase the replication factor for the in-demand data once a particular data becomes popular. On the contrary, if the data losses its popularity over time, replica management frameworks adapt the replication factor back to the default level.

Changing the replication factor also changes the block distribution on the cluster. The influence of increasing the replication factor has been widely studied [9, 15, 16]. However, our previous work [17] was the first to identify that the current replica deletion algorithm of Hadoop can be the cause of performance degradation. Consequently, we proposed Workload-aware Balanced Replica Deletion (WBRD). WBRD achieves up to 48% improvement in job completion time compared to HDFS by balancing the number of stored blocks for a particular data-set rather than the disk usage in each node [17]. WBRD’s even block distribution strategy does not take nodes’ processing capabilities into consideration. However, current Hadoop clusters are highly scaled systems and composed of numerous racks (set of nodes) and generally, each rack contains nodes with the same characteristics. Racks can be upgraded or replaced separately. Hence, heterogeneity occurs in highly scaled Hadoop clusters [18]. WBRD is limited and results in sub-optimal performance for the case of heterogeneous Hadoop clusters.

In this paper, we propose a novel cost-effective Heterogeneity-aware Replica Deletion algorithm (HaRD) to cover the case of heterogeneous clusters. The primary goal of HaRD is to balance the ratio of block distribution to the computing capabilities for each node. Therefore, HaRD tries to enhance the system by placing more blocks in powerful machines. HaRD determines the computing capability of each node by calculating the number of containers it can run simultaneously. We implemented HaRD on top of HDFS and conducted a comprehensive set of experiments with representative benchmarks to evaluate the performance of HaRD against WBRD, as well as Hadoop. Experimental results on a heterogeneous 23 nodes Hadoop cluster show that HaRD speeds-up the system performance for the single query, and reduces execution time by 40% and 8% on average when compared to HDFS and WBRD, respectively. Moreover, improvements become more compelling when the system is highly-utilised by a large number of concurrent requests, and increase to 60% and 17% compared to HDFS and WBRD, respectively. The present study makes the following contributions:

1. We show the current replica deletion algorithms (both Hadoop and WBRD) do not consider the processing capability of nodes, and thus heterogeneous clusters become an edge case.
2. We extend the formal definition of the replica deletion problem to heterogeneous clusters.
3. We propose a novel cost-effective Heterogeneity-aware Replica Deletion algorithm (HaRD). In order to consider heterogeneity in the cluster, HaRD uses a container-based approach to calculate the computing ratio of each machine.
4. We implement the proposed approach and evaluate both the performance improvement and its overhead by conducting an extensive set of experiments on a heterogeneous 23-node Hadoop cluster.

The remainder of this paper is organised as follows: "Background" section provides background information. The related work is reviewed in "Related work" section. "Improving performance of replica management system through heterogeneity-aware replica deletion" section identifies the replica deletion problem and models the problem in the context of heterogeneous clusters and details novel HaRD algorithm. "Methods" section describes the experimental environment. "Results and discussion" section presents the results of our evaluation. Finally, "Conclusion" section concludes this paper.

## Background

HDFS [6] is one of four core modules of the Hadoop Project [4] and is responsible for storing data in a distributed fashion. The design principle behind HDFS is to develop a distributed mass-storage system as a main pillar for the Hadoop ecosystem [6]. Therefore, HDFS is highly scalable and capable of storing tremendous data-sets on a large number of commodity machines. On such a scale, node failures are more than a theoretical probability and can occur for various reasons, e.g., hardware failure, power losses. Hence, HDFS’s architecture strengthens fault-tolerance by benefiting from the technique of replication and distributed storage of replicated data.

HDFS has a master-slave model and is composed of two primary daemons: NameNode (NN) and DataNode (DN). NameNode, the master, is responsible for storing meta-data and operations related to meta-data. NN keeps track of DNs by checking their heartbeat messages periodically. When a DN fails or becomes unavailable, the NN marks it as dead and coordinates data re-replication. Moreover, the NN manages data requests and directs them to relevant DNs. DataNode, the slave, is responsible for storing blocks and serving blocks for data requests. The number of DNs can easily scale to thousands and can store tens of petabytes [6].

**Fig. 1:**
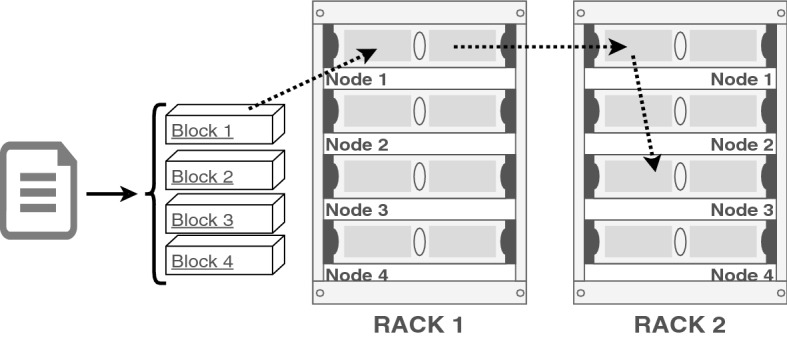
Data uploading to the cluster

HDFS organises the stored files in a traditional, hierarchical file structure. The main directory of the system, *Root*, is at the top of the hierarchy. Therefore, any stored file on HDFS is a part of *Root*’s branches. While uploading the data into a Hadoop cluster, first, data is divided into fixed-size blocks. The fixed block size is 64 MB in Hadoop 1; however, it has been increased to 128 MB in the Hadoop 2. The blocks are replicated three times by default and placed among the nodes in the cluster. In order to place replicas over different nodes, HDFS leverages a data pipeline rather than using one centralised node to transfer all of the replicas. In the pipeline, replicas are passed from one DN to another DN as shown in Fig. 1. This decentralised strategy improves the efficiency of the replica transmission by sharing the network load among the nodes and reduces the chance of a possible network bottleneck.

Block placement is performed according to Hadoop’s data placement policy [19]. The policy prefers to place the first replica into the DN that sent the request (the client), otherwise it is put on a DN that is on the same rack with the client node [19]. The second replica is placed on a node that is on a different rack from the first replica. The last (third) replica is placed on the same rack as the second replica but a different node. The default placement policy is rack-aware as it tries to place replicas into at least two different racks in the case of multiple rack environment. There are two advantages of using the rack-aware replica placement. Firstly, it enhances the fault-tolerance of the system. Thus, submitted jobs can be completed even if a rack fails during the execution. Secondly, the default placement algorithm benefits from having multiple racks and improves network usage by reducing off-rack traffic. The reason for this is the network traffic is significantly faster between nodes on the same rack than on different racks.

**Fig. 2:**
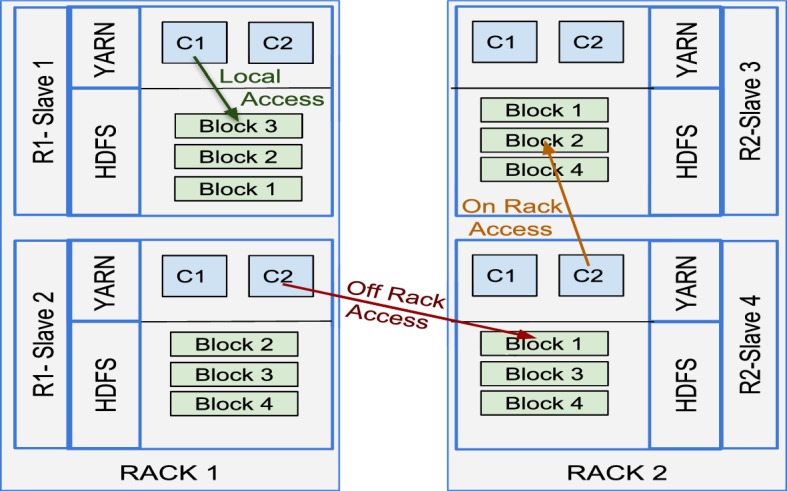
Data locality types in Hadoop jobs

Data locality means processing data where that data is stored and is the fundamental idea behind data-intensive computing. In data-intensive computing, data-sets are immense and thus, moving the data from one machine to another requires significant network traffic. Conversely, the code that needs to be executed is much smaller than the data itself. Therefore, moving the computation to the data is easier than the opposite. The strategy, “moving computation is cheaper than moving data” [4], is employed by HDFS to improve the efficiency of the system. When a job is submitted to the Resource Manager, the job is first divided into smaller tasks. Then, each task is associated with a split (i.e., a specific portion of data). Most of the time, the splits are created based on the HDFS block size. However, this is not always the case, as it completely depends on the job’s *getSplits* method. Created splits are associated with map tasks. Hadoop prefers to schedule split-associated map tasks on the node that keeps the split. Moreover, Hadoop can even delay the start of tasks to reach better data locality [20]. Any map tasks that can not be scheduled to run in data local mode require extra data transmission, increasing the network utilisation; consequently, increasing the total execution time. There are three different task execution type for the data locality as shown in Fig. 2:

- Local access: the same node stores the data and executes the task, e.g., R1-Slave 1 needs to process Block 3.
- Same rack access: the processing node does not store the data split and requests it from another node that is located on the same rack in order to start the task, e.g., when R2-Slave 4 needs to process Block 2, it requests the block from R2-Slave 3 (which is in the same rack).
- Off rack access: the processing node does not store the data split and requests from a node that is located on another rack, e.g., when R1-Slave 2 needs to process Block 1, it requests the block from R2-Slave 4.

In the event that running a task in local access mode is not possible after multiple attempts, Hadoop’s task scheduler gives priority to running the task on a node that is located on the same rack where data is stored. The reasoning behind is the same as the benefits of using multiple racks, the network traffic between nodes on the same rack is significantly faster compared to the nodes on different racks. Therefore, the scheduler exploits on-rack access rather than off-rack to reduce slow inter-rack traffic. The worst case scenario is the last option of task scheduling, allocating a node that is on a completely different rack and that requires off-rack access.

## Related work

Even though large-scale Hadoop clusters can store a tremendous amount of data, the demand for each stored data-set is not the same. Moreover, the data-set demand changes over time. Hence, several studies have been conducted to understand the workload of Hadoop clusters [11, 21]. Ananthanarayanan et al. [11] underlined that 12% of the most popular files are more in demand and received ten times more requests than the bottom third of the data (based on the analysis they have accomplished from logs of Bing production clusters). Another study [21] was conducted by analysing three different workload traces (i.e., OpenCloud, M45, WebMining) with various cluster sizes (from 9 nodes to 400 nodes). The authors [21] draw attention to load balancing problems in the Hadoop cluster. Furthermore, the same study showed that despite the data distribution being well-balanced, the task distribution remains unbalanced. Consequently, an unbalanced cluster leads to poor data locality and performance degradation for the cluster.

Data replication is a prominent method to improve fault-tolerance and load-balancing [9, 15, 16]. However, increasing the number of copies stored in the cluster comes with the price of extra storage. Considering the fact that not all data-sets have the same demand, there is no one-size-fits-all solution for the replication factor. Therefore, various approaches have been proposed in the literature for adapting the replication factor according to the access pattern of data-sets [11–14, 22]. All of these strategies alter the replication factor either proactively [11] or dynamically [12–14, 22] based on the ‘hotness’ of the data. Wei et al. [12] propose a cost-effective dynamic replication management scheme for the large-scale cloud storage system (CDRM). With the intention of developing such a system, the authors built a model between data availability and replication factor. Ananthanarayanan et al. [11] present Scarlett for adapting the replication factor by calculating a storage budget. Abad et al. [13] propose an adaptive data replication for efficient cluster scheduling (DARE). DARE aims to identify the replication factor dynamically based on probabilistic sampling techniques. Cheng et al. [14] introduce an active/standby storage model and propose an elastic replication management system (ERMS) based on the model. ERMS places new replicas of in-demand data to active nodes in order to increase data availability. Lin et al. [22] approach the problem of adapting the replication factor from an energy-efficiency perspective and propose an energy-efficient adaptive file replication system (EAFR). EAFR places ‘cold’ files into ‘cold’ servers to reach energy efficiency.

In addition to adapting the replication factor, the placement of blocks is another factor to achieving good load-balancing. Eltabakh et al. [15] propose CoHadoop to co-locate related files based on the information gathered from the application level. CoHadoop leverages data pre-partitioning against expensive shuffles. Xie et al. [23] and Lea et al. [24] propose placing blocks based on the computing ratio of each node. Liao et al. [25] describe a new approach to the block placement problem based on block access frequency. The authors investigated the history of block access sequences and used the k-partition algorithm to separate blocks into different groups according to their access load. Moreover, the placement in hybrid storage systems [26, 27] and smart caching approaches for remote data accesses [28] is also proposed in the literature. There is a considerable amount of research about the block placement because the block placement is decisive for the system performance. However, the connection between replica management systems and the block placement is missing. For instance, which replica should be deleted when the framework decides to reduce the replication factor? One simple approach would be to use HDFS’s deletion algorithm.

But altering the replication factor changes the block density on each node. The framework that adapts the replication factor should also be aware of how the replicas are distributed. Otherwise, the cluster ends up with unbalanced data distribution and consequently unbalanced load distribution. In our previous work [17], we identified that decreasing the replication factor leads to data unbalancing in HDFS and we proposed Workload-aware Balanced Replica Deletion (WBRD) to balance the data-set distribution among the nodes. As a result, WBRD achieves up to 48% improvement in execution time on average. But, WBRD does not fully exploit different nodes’ processing capability as it is designed for homogeneous clusters. One approach to determine nodes’ processing capability is to measure computing ratios for each different application on each node [23, 24]. However, as the workload of the cluster is highly dynamic and contains multiple ad-hoc queries, we prefer to use a more flexible and cost-effective approach. Therefore, instead of following previous approaches, the present work employs a novel cost-effective container-based approach.

## Improving performance of replica management system through heterogeneity-aware replica deletion

### Replica management

Files stored on HDFS are replicated according to the cluster’s default RF value configuration: *dfs.replication*. However, the replication factor (RF) is a file-level setting; different values can be set for different files. Moreover, the RF can be altered anytime after the creation of a file through the command: *hadoop fs -setrep [-R] [-w]* <*numReplicas*$> <$*path*>. Keeping more copies of files increases data availability and the chance of running tasks in data-local mode. Hence, replication provides better load-balancing, data locality and ultimately, reduces jobs’ execution time. Since a tremendous amount of data is stored on a data-intensive cluster, keeping a few extra copies for all of the data is clearly an extravagant solution.

Consequently, replication management frameworks were proposed to identify the ‘best’ RF for each file individually to achieve better performance while minimising the extra storage overhead of increasing replication factor. In addition to identifying the RF, placing these replicas is another crucial problem. Even though all of the proposed replica management frameworks strive to enhance the performance, adapting the RF changes the block distribution. If replica creation/deletion algorithms do not consider balancing the data-set; they end up with a skewed (unbalanced) distribution.

Typically, even block distribution helps to utilise all nodes equally during the execution of tasks in the cluster and performs better compared to the skewed distribution in homogeneous clusters. In the case of skewed data distribution, some nodes keep more data than others. Consequently, these nodes can transform into a ‘hot spot’ of the cluster as the data needs to be constantly transferred from hot spots to other nodes during jobs’ processing. Thus, data locality decreases, network utilisation burgeons and processing takes more time due to the waiting time that occurs in data transmission.

In our previous work [17], we already showed the current deletion algorithm in Hadoop does not perform well and consequently proposed a workload-aware balanced replica deletion algorithm. The deletion algorithm in Hadoop only concerns itself with balancing the overall cluster. More importantly, it does not update the state of utilisation metrics after each deletion. Unlike HDFS default policy, WBRD aims to balance data-sets distribution rather than the overall cluster and achieves better performance. The purpose of the present study is to highlight the limitation of WBRD for a heterogeneous cluster and to propose Heterogeneity-aware Replica Deletion (HaRD) to address the shortfall of WBRD.

#### Motivational example

**Fig. 3:**
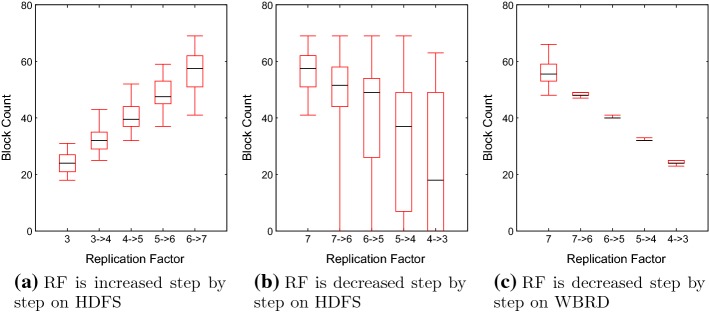
The block distribution when the replication factor (RF) is altered

In this section, we would like to illustrate the replica deletion problem empirically and discuss the limitation of WBRD to motivate the work. Figure 3 shows the evolution of block distribution on the 23-nodes Hadoop cluster while the RF is altered. More particularly, Fig. 3a, b reports the block distribution by using default (HDFS) placement policy when the replication factor is increased and decreased, respectively. On the other hand, Fig. 3c reports the block distribution during the replica deletion by using WBRD.

When the replication factor is increased as shown in Fig. 3a, the number of blocks that are stored on each node varies. However, the range is small and thus, each node stores a similar number of blocks. Therefore, the standard deviation (SD) is not substantial and leads to the narrow inter-quartile range. As a result, the block distribution is well-balanced. On the contrary, Fig. 3b presents the distribution when the RF is decreased. After the first deletion, the block distribution range starts from zero which means at least one of the nodes does not participate in data storage. Moreover, the maximum value of the range stays dominantly the same and shows that at least one of the nodes keeps the majority of the data. Consequently, the overall range increases and also inter-quartile range increases and leads to imbalanced data distribution.

Figure 3c shows block distributions when the replication factor is reduced by using WBRD. Unlike the HDFS deletion approach, WBRD tries to balance the overall data-set during the replica deletion. Therefore, we can see the inter-quartile range is small and does not vary. Subsequently, WBRD achieves greater performance compared to default HDFS. Albeit, WBRD is limited as it does not consider processing capabilities. If each node has different processing capabilities (e.g., heterogeneous clusters), an even block distribution would not be an optimal case for efficiency. In such a scenario, powerful nodes finish their tasks before slower nodes. As a result, either the task in the scheduler queue waits for slower nodes until slower nodes become available for processing while powerful nodes are idle or data needs to be transferred from slower nodes to powerful nodes in order to continue processing. In both cases, jobs are delayed (considering the fact that the job is only completed when all sub-tasks are processed fully).

Hence, WBRD’s even distribution performs sub-optimally, and heterogeneous clusters become an edge case. With the intention of improving the performance even further, our hypothesis is to keep more replicas on the nodes that have more processing capabilities; therefore, the workload distribution would be more balanced. We modelled the replica deletion problem in the context of heterogeneous clusters and proposed heterogeneity-aware replica deletion algorithm to achieve the modelled objectives.

### Formal definition

We assume a cluster ${\mathcal {C}}$ is composed of a set of racks *Racks*. Each rack $Rack_n \in Racks$ contains a set of machines $m\in {\mathcal {M}}$ such that *Rack*(*m*) = $Rack_n$. A set of files ${\mathcal {F}}$ is stored in the cluster ${\mathcal {C}}$ over the machines $m\in {\mathcal {M}}$. Every file $F_i \in {\mathcal {F}}$ is divided into fixed-sized blocks $B_{i}$ (128 MB by default) as stated in Eq. (1) and stored in a hierarchical file organisation.

1 $$\begin{aligned} |B_i| = \left\lceil \frac{\text {File Size}}{\text {Block Size}}\right\rceil \end{aligned}$$

Root path, *Root*, is the ‘highest’ level of the hierarchy and every file $F_i \in {\mathcal {F}}$ is placed into a certain path *P* which is a branch of *Root*. Each block $b_{ij} \in B_{i}$ is replicated ${RF}_{i} \in \mathbb {N}^{*}$ times (i.e., the replication factor of file $F_i$). We denote $b_{ij}^{u}$ the replica number *u* of the block $b_{ij}$ where $0< u =< {RF}_{i}$. Each replica $b_{ij}^u$ is stored a particular machine $M(b_{ij}^u)$.

We want to reduce the replication factor from $RF_i$ to $RF'_i$ such that $RF_i > RF'_i$. We introduce a binary variable $x_{ij}^{u}$ which takes the value 1 if the replica *u* of the block $b_{ij}$ exists after reducing the replication factor, or 0 otherwise.

2 $$\begin{aligned} \sum _{u=1}^{RF_i} x_{ij}^{u} = RF'_{i},~~ \forall ~ F_i \in P, ~~\forall ~j \in \{1,...,|B_i|\} \end{aligned}$$

To strengthen fault-tolerance and promote data availability, replicas are distributed over different racks according to a rack-awareness condition in the default block placement policy as expressed in Eq. (3). The proposed algorithm continues the rack-awareness block placement after a successful deletion for $\forall ~ F_i \in P, ~~\forall ~j \in \{1,...,|B_i|\}$:

3 $$\begin{aligned} \left| \left\{ Rack(M(b_{ij}^u) )~|~u \in \{1,...,RF'_{i}\}~and~x_{ij}^u = 1~\right\} \right| \ge 2 \end{aligned}$$

We defined a variable for the partial block count $PBC_m \in \mathbb {N}$ for each machine $m \in {\mathcal {M}}$. For a given path, $PBC_m$ is computed as a sum of all replicas for $\forall ~ F_i \in P$ that are stored on the machine *m* as expressed in Eq. (4):

4 $$\begin{aligned} PBC_m = ~\sum _{i \in \{1,..., |F_i|\}}~\sum _{j \in \{1,..., |B_i|\}}\sum _{ \begin{array}{c} u \in \{1,...,RF_i\} \\ \wedge ~M(b_{ij}^u)=m \end{array} } x_{ij}^{u} \end{aligned}$$

Each *m* has finite resources: (e.g., CPU and RAM) denoted by *vCore*(*m*), *RAM*(*m*) respectively. The network connection between machines $m_i, m_j\in {\mathcal {M}}$ in the same rack (i.e., $Rack(m_i) = Rack(m_j)$) is faster compare to machines are in the different rack (i.e., $Rack(m_i) \ne Rack(m_j)$). Any submitted job (a.k.a., task) runs on a container allocated to particular node *m* with resource requirements $vCore_{Cont}$ and $RAM_{Cont}$. Note that $vCore_{Cont}$ and $RAM_{Cont}$ both are global properties of the scheduler [29]. A machine $m\in {\mathcal {M}}$ can run a number of containers ${\mathcal {K}}_m$ concurrently. ${{\mathcal {K}}_m}$ is determined by composition of available machines’ resources (i.e., *vCore*(*m*) and *RAM*(*m*)) and containers’ resource requirements (i.e., $vCore_{Cont}$ and $RAM_{Cont}$) as expressed in Eq. (5):

5 $$\begin{aligned} {\mathcal {K}}_m = \min \left( \left\lfloor \frac{\textit{RAM}(m)}{\textit{RAM}_{Cont}}\right\rfloor , \left\lfloor \frac{\textit{vCore}(m)}{\textit{vCore}_{Cont}}\right\rfloor \right) \end{aligned}$$

While deleting replicas, our main objective is to minimise the maximum ratio of ${PBC_m}$ to ${{\mathcal {K}}_m}$ for a given path $P \subseteq {\mathcal {P}}$ as shown in Eq. (6). Therefore, in every deletion iteration our algorithm will select a replica that has the biggest division value. Hence, we expect to see the number of replica stored on a node become dependent on ${{\mathcal {K}}_m}$ after our replication deletion algorithm.

6 $$\begin{aligned} minimise~ \max _{m\in {\mathcal {M}}}\left( \frac{PBC_m}{{\mathcal {K}}_m}\right) \end{aligned}$$

### Heterogeneity-aware replica deletion (HaRD)

To address the problem detailed in "Motivational example" section, we propose Heterogeneity-aware Replica Deletion (HaRD) as shown in Algorithm 1. The primary objective of HaRD is to attain a uniform distribution of the ratio of the block distribution to computing resource for a given path while satisfying the stated constraints. HaRD starts with the determination of computing capabilities ${\mathcal {K}}_m$ of each machine. One existing approach to define the computing ratio is to measure the performance of each job in each node [23, 24]. However, measuring the performance of each job on different types of nodes is not efficient as multiple users concurrently query the system with various ad-hoc queries in real Hadoop clusters. Therefore, we put forward a new approach to determine the computing capability (ratio) of each node through how many containers can run simultaneously on each node manager. After the establishment of YARN (announced in Hadoop 2.0), submitted jobs in a Hadoop cluster run on the container that is allocated by the node manager. The main idea of YARN is to bring flexibility to the map/reduce task scheduling which was statically defined in Hadoop 1.0. The resource manager of YARN organises the allocation of containers by coordinating with node managers and schedules an application based on a node’s resource usage. Consequently, computing ratios can be used as expressed in Eq. (4). Our YARN-based approach provides flexibility and extensibility since new processing features (i.e., the use of GPUs in the Hadoop cluster is becoming mainstream [30]) are implemented on the top of YARN. We would like to underline that HaRD is based on YARN and therefore, creates an minimal overhead. We are aware that our present work depends on a correct YARN configuration. Such an assumption is not a strict constraint as YARN is generally configured during the deployment process [29, 31].

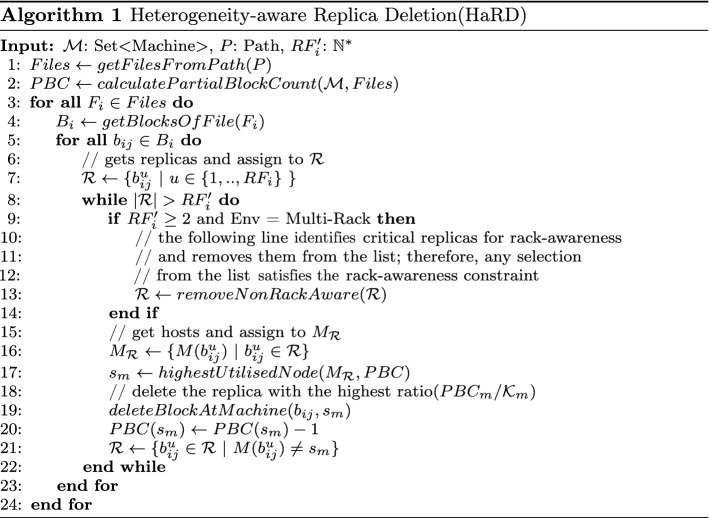

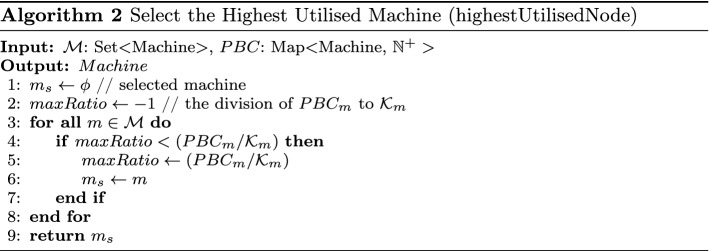





After ${\mathcal {K}}_m$ is determined, HaRD can be used to decrease the replication factor. When a user or a replica management framework alters $RF_i$ to $RF'_i$ such that $RF_i > RF'_i$ for a particular path, then HaRD will be executed. HaRD starts with the calculation of $PBC_{m}$ for each node in the cluster. For this, HaRD retrieves the replica list by iterating every block of files stored on a *P*. In the case that an environment is multi-rack, HaRD uses *removeNonRackAware* method to remove the set of replicas that violate rack-awareness constraints. Therefore, HaRD ensures the state after the deletion satisfies rack-awareness constraints. HaRD scans through every replica in the list *R* and finds the replica that is stored on the most-utilised node by comparing the ratio of ${PBC_m}$ to ${{\mathcal {K}}_m}$. Finally, it removes replicas from the list by using the method *deleteBlockAtMachine*. Deletion iterations run for all blocks of each file. If the data distribution is uniform at the beginning, HaRD starts deletions from the least powerful nodes (i.e., ${min ({\mathcal {K}}_m})$). After a few iterations, HaRD balances the nodes’ ratio of ${PBC_m}$ to ${{\mathcal {K}}_m}$. Then, the rest of the deletion iterations continue by maintaining the ratio until the last data block is processed.

We would like to note that the value of ${{\mathcal {K}}_m}$ would be the same for every node if the cluster is homogeneous. In such a case, HaRD works in the same way as WBRD. For homogeneous clusters, we already found that WBRD achieves up to 48% improvement in execution time when compared to HDFS [17]. Therefore, our experiments in this paper do not consider the case of homogeneous clusters.

#### Implementation

Whenever the replication factor is altered for a path, the deletion request is made to the NN by calling the *setReplication* method in *FSNamesystem.java* with a path and a number of replicas. If the NN is not in safe mode, the requested operation is started by invoking setReplication method in *FSDirAttrOp.java*. The method returns true if the operation completed successfully or return false if any problem occurs during the operation. If $RF_i$ is less than $RF'_i$ (i.e., the replication factor is increased) in *setReplication* method, the order for allocating new replica is placed into the priority queue of under-replicated blocks. On the contrary, if $RF_i$ is bigger than $RF'_i$ (i.e., the replication factor is decreased), then *processOverReplicatedBlock* is executed for the replica deletion. In order to select the next replica for the deletion, the method collaborates with *chooseReplicasToDelete* method in the class of block placement strategy.

Hadoop supports the use of customised block placement policies by including a pluggable interface for the block placement [19]. For this reason, Hadoop contains fundamental methods for the placement which is in the abstract pluggable policy. We implemented HaRD by modifying the source code of HDFS on the top of Hadoop (version 2.7.3). To implement the proposed deletion strategy, we first created a new block placement policy for HaRD by inheriting the existing block placement policy. Then, we overrode the method of *chooseReplicasToDelete* in HaRD’s placement strategy. Moreover, we also modified the block manager class to retrieve ${{\mathcal {K}}_m}$ and pass it to HaRD’s placement policy.

We prefer to use the pluggable block placement policy; thus the placement policy can be changed by altering *dfs.block.replicator.classname* the configuration in *hdfs-site.xml* without changing the source code. We are aware that HaRD’s implementation brings extra operations and can lead to overhead on the system. However, the all of the newly implemented code is only executed in the case of replica deletion occurs. Otherwise it will not have impact on the system performance. We evaluated the overhead using different data-set sizes as well as different number of nodes. The scalability of HaRD is discussed in "Overhead analysis" section.

## Methods

Our experiments were conducted on the Performance Engineering Laboratory’s research cluster (in University College Dublin). The cluster consists of 23 dedicated machines (1 master and 22 slaves). In this cluster, 20 slaves are identical. Therefore, we used cgroups, Linux kernel feature, to limit computing resources to create heterogeneity in the cluster. We limited 10 nodes’ CPU to 2 virtual cores and RAM to 4 GB. Overall, the cluster is composed of 3 different types of nodes. We detailed the resource specification in Table 1 for each type of machines. All nodes are equipped with 1 TB hard-drive and connected with a Gigabit Ethernet switch.

**Table 1:** Resource specifications for the cluster

Computer set	CPU type	Allocated VCore	Allocated ram (GB)	Number of machines
Master	i7-6700	8	32	1
Slaves-1	i5-6500	4	8	10
Slaves-2	i5-6500	2	4	10
SlavesXL-1	Xeon E5-2430	12	48	2
Total		92	248	23

The operating system selected was Lubuntu, which runs on kernel Linux 4.4.0-31-generic, and the java version 1.8.0_131 was installed. All tests were run on Hadoop version 2.7.3 (native, WBRD and HaRD). Hive version 1.2.2 was selected for concurrency tests on TPC-H. Ganglia [32] was used for monitoring the cluster.

### Testing methodology

In this section, we detail the testing methodology for the experiments. Each test starts with a new Hadoop cluster deployment. After the successful deployment, the benchmark’s data-set is uploaded to the cluster. It is important to note that both TestDFSIO and Terasort benchmark suites can create their data-set with any given size. So, we populated them only one time and we repeated tests by using the same data-set during experiments of both TestDFSIO and Terasort. Hence, we ensured the input is the same for all algorithms under-test. After the data loading phase, we increased the replication factor from 3 to 10; consecutively, decreased to three unless otherwise stated. We would like to note that even though we used 10 as a higher replication factor, any value above 3 creates the similar distribution. Every benchmark is run ten times for statistical significance. We normalised results of execution time by using the average of these runs. The plotted graphs presented indicate the range of results.

### Benchmarks

Hadoop tasks can have different bottlenecks: excessive usage of disk I/O, network utilisation, or CPU utilisation. To carry out a reliable test, we selected three well-known benchmarks [33, 34]. Each benchmark has different characteristic and focuses on stressing different part of the system and all comes out-of-the-box with Hadoop release: (i) TestDFSIO, (ii) Grep and (iii) Terasort. Hadoop clusters are large-scale distributed and multi-tenant systems. Therefore, numerous queries can be executed by many users at the same time. The usage of query-like frameworks is common in the production as highlighted by previous studies [35, 36]. Hence, in addition to three popular benchmarks, we include the concurrency test on Hive [37] to represent production domains and test concurrency.

#### TestDFSIO

TestDFSIO is a well-known benchmark to measure the distributed I/O throughput of HDFS. TestDFSIO stresses the disk performance and reports both read and write performance of the system. The benchmark is a highly representative test for tasks that suffer from I/O bottlenecks. During the experiment, we reported only the reading part of the test, since we assessed the effect of data distribution on the reading performance. To be fair in each test case, first, we populated a 100 GB data-set by using the benchmark’s write functionality and conducted reading throughput tests by using the same data-set.

#### Grep

Grep is another standard Hadoop benchmark and evaluates the system performance by searching and counting the number of times a given keyword appears in the text. The benchmark has a read-intensive characteristic and also stresses CPU by sorting data. Grep runs two jobs sequentially, the first job calculates the number of times a matching string appears and passes it to the second job. The second job sorts the result of the first job according to the matching string’s frequency. We run the Grep benchmark on the NOAA data-set from the National Centres for Environmental Information. The data-set was composed of 8 years collected data (between 2008/05–2016/04, 47.3 GB). In our test, we were looking for data that is generated in January 2011 as a condition; thus, the keyword was chosen as ‘2010,1,’.

#### Terasort

Terasort is a well-known standard benchmark used to stress the whole system. The benchmark assesses the performance of Hadoop clusters by sorting data. The task is not only read-intensive but also network-intensive as it requires expensive data shuffles while passing data from map tasks to reduce tasks. We created a 50 GB data-set by using TeraGen and used the same data-set to test each algorithm.

#### Concurrency test on TPC-H

TPC-H is a decision support benchmark and in use for assessing the performance of relational databases [38]. The purpose of including tests with TPC-H is SQL-on-Hadoop systems (e.g., Hive [37], Impala [39], VectorH [40]) has brought the comfort and flexibility of SQL to Hadoop for querying ‘big’ data; thus, SQL-on-Hadoop has become mainstream in the industry for big data analytics [41]. To represent the SQL-on-Hadoop domain, we conducted the concurrency test on a 30 GB TPC-H data set. The concurrency test has been performed on Hive version 1.2.2 with different numbers of users: *{25,50,75,100,125}* and a 1-second interval between each query run by using Q6.

## Results and discussion

We evaluated the performance of HaRD on the 23-node heterogeneous Hadoop cluster through conducting the following experiments: (i) analysed the data distribution after the replica deletion with three different data-set sizes: {64 GB, 128 GB, 256 GB}, (ii) executed three different well-known benchmarks with various replication factors, (iii) performed concurrency test on TPC-H with numerous concurrent users: {25, 50, 75, 100, 125}, and (iv) conducted in depth-analysis to understand improvements in the aspects of data locality and network utilisation.

### Block distribution

**Fig. 4:**
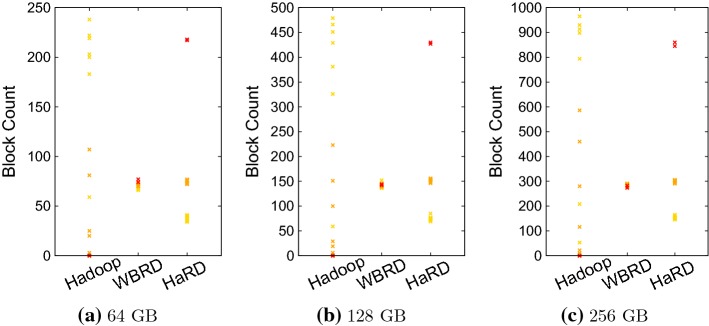
Block distribution after the RF is decreased back to 3

Performance of distributed systems is highly dependent on how the data is distributed among the nodes. Additionally, it is even more important if the distributed system is running many data-intensive jobs. Therefore, our first comparison is the block distribution. Figure 4 reports the comparison of block distribution after the RF is reduced from 10 to 3 by using the different deletion algorithms. Each cross-mark in the figures indicates the number of blocks that is stored on a particular node. Since the cluster is composed of three different types of nodes (namely, Slaves-1, Slaves-2 and SlavesXL-1), we used marks with three different colours to demonstrate the processing capability of nodes. Colour yellow, orange and red identify the marked node belongs to Slaves-1, Slaves-2 and SlavesXL-1, respectively. It can be seen from the figure that Hadoop’s deletion algorithm has high SD for the spread of numbers blocks per node (91.3 for 64 GB, 182.1 for 128 GB and 368.6 for 256 GB) compared to mean value (74.2 for 64 GB, 141.8 for 128 GB and 283.6 for 256 GB) and causes skewed data distribution in every case, as it tries to balance overall cluster’s disk utilisation. Unlike Hadoop, WBRD tries to balance ${PBC_m}$ for every node in the cluster. Thus, WBRD achieves the evenly balanced block distribution with the low SD (2.3 for 64 GB, 3.4 for 128 GB and 4.9 for 256 GB) compared to mean value (70.9 for 64 GB, 141.8 for 128 GB and 283.6 for 256 GB); however, the even block distribution is not fair in terms of the workload distribution since heterogeneity exists. Consequently, WBRD causes the unbalanced workload in the heterogeneous cluster. On the other hand, HaRD aims to balance the ratio of ${PBC_m}$ to ${{\mathcal {K}}_m}$ for every node in the cluster. Thus, HaRD stores more blocks on more powerful computers; it creates three different groups in the block distribution as the cluster is composed of three different machine types.

### Average execution time

**Fig. 5:**
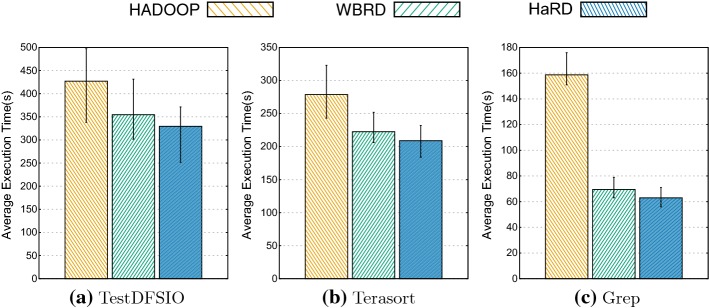
Test benchmarks with RF: 3

We conducted our experiment by using three fundamental well-known benchmarks and compared the performance of each algorithm according to their execution time on average. Figure 5 presents the result of three different benchmarks (namely, TestDFSIO, Terasort and Grep) with the RF of 3 for three different deletion algorithms. During experiments, we observed that WBRD achieves notable improvements against Hadoop. However, the system performance enhances even further with HaRD due to the balanced workload distribution as the computing capability of each node is taken into account during the replica deletion. As a result, HaRD reduces average execution time 7% for TestDFSIO, 6.1% for Terasort and 9.4% for Grep compared to WBRD. When we compared the performance of HaRD against HDFS, the improvements become remarkable: 60.3% for Grep, 22.8% for TestDFSIO and 25% for Terasort. Even though each test benchmark has a different bottleneck; HaRD consistently performs best in all tests.

**Fig. 6:**
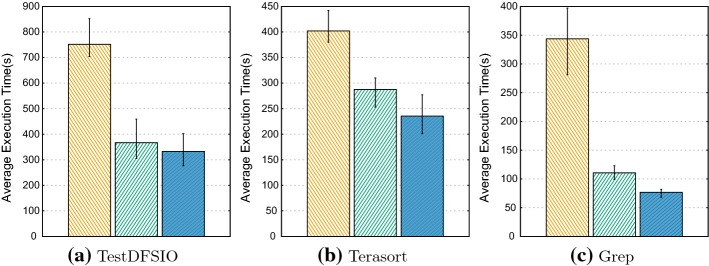
Test benchmarks with RF: 1

While we were conducting our experiments, we also focused on the performance evaluation under lower the RF value due to the strong dependency between RF and job execution time. The impact of having more replicas has a significant effect on the performance when the system scales [9]. So, tests with the RF:1 acts as tests on bigger clusters. Figure 6 shows the result of the same test benchmarks but this time with the RF of 1. Similar to the results of performance tests with the RF of 3, HaRD outperforms both WBRD and Hadoop with a single replica. HaRD reaches better performance by reducing job execution time: 18.1% for TestDFSIO, 9.2% for Terasort and 30.6% for Grep compared to WBRD. Moreover, the performance gain of HaRD over default Hadoop, in terms of execution time, is 55.7% for TestDFSIO, 41.4% for Terasort and 77.6% for Grep.

### Testing with concurrent users

**Fig. 7:**
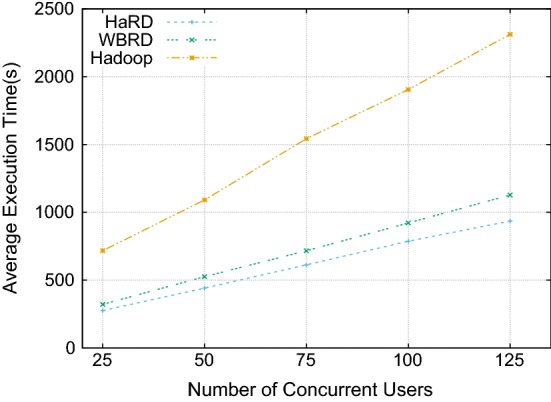
Concurrency test with TPC-H Q6

Hadoop clusters are designed to serve as multi-tenant systems, and the cluster is queried by numerous users at the same time. Therefore, we include the concurrent user test by using TPC-H Q6. Figure 7 reports the average execution time for the concurrent users test and demonstrates that Hadoop performs worst in every case and also shows HaRD performs better than WBRD. Improvements of HaRD compared to WBRD in job’s execution time starts from 14% for 25 concurrent users and increases up to 17% as we stress the system with more concurrent users. Furthermore, the enhancement in execution time is around 60% for the all different number of users compared to default HDFS. We want to note that there is no difference observed between HaRD and WBRD while testing with single TPC-H queries since single queries do not fully stress the system; but, both HaRD and WBRD still perform significantly better than Hadoop. This experiment underlines that the performance improvements become more significant when the system is fully utilised under the heavy load of concurrent users.

### In-depth analysis

**Fig. 8:**
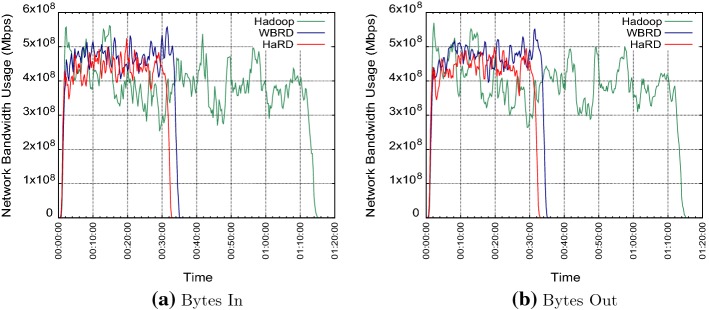
Network bandwidth usage during the test with 125 concurrent users

We performed an in-depth analysis of the 125 users concurrency test to understand and observe improvements in data locality and network utilisation. The system is monitored by using Ganglia for the network metrics and Hadoop’s HistoryServer for the data locality during the experiment. We measured the data locality by $\dfrac{|Data Local Tasks|* 100}{|All Tasks|}$. For 125 concurrent users, we found that approximately 85% of all jobs are data-local for HaRD; however, the data locality drops to 81% for WBRD and 73% for Hadoop Jobs. So, WBRD transfers 376 more splits during the test compared to HaRD. Running more data-local jobs reduces the number of blocks that need to be transferred during execution and in turn leads to less network usage. We inspected the network bandwidth usage and plotted network graphs in Fig. 8. Fig. 8a, b shows the aggregated network utilisation both bytes in and bytes out, respectively. Average network bandwidth usage is 402 Mbps for HaRD, 432 Mbps for WBRD and 378 Mbps for Hadoop. We can see the proposed algorithm, HaRD, reduces the average network utilisation approximately 30 Mbps (6.9%) compared to WBRD. When we compared three algorithms for the overall network usage, Hadoop performs worst due to the high execution time. Interestingly, Hadoop has the lowest average network utilisation even though it has the lowest value for the percentage of data-local jobs.

To understand default Hadoop’s network behaviour, we carried out further investigation by observing the network usage on each node individually. We found that the data is in a continuous flow from ‘hot spots’ to other nodes due to the fact that the majority of blocks were located on ‘hot spots’. Thus, we can see higher values for data out on ‘hot spots’ and lower data out values on other nodes. Conversely, this trend is opposite for data in; data in is low on ‘hot spots’ and high on the rest of nodes. Therefore, the network utilisation is not well-balanced on the cluster. Moreover, Hadoop’s network bandwidth usage is not stable due to the high SD of the block distribution; but more importantly, reaches higher peaks compared to WBRD and HaRD. On the contrary, we identified that the network bandwidth usage in both WBRD and HaRD is balanced on each node. The results show that the default Hadoop deletion algorithm causes an imbalance in the network bandwidth usage in the cluster.

### Overhead analysis

**Fig. 9:**
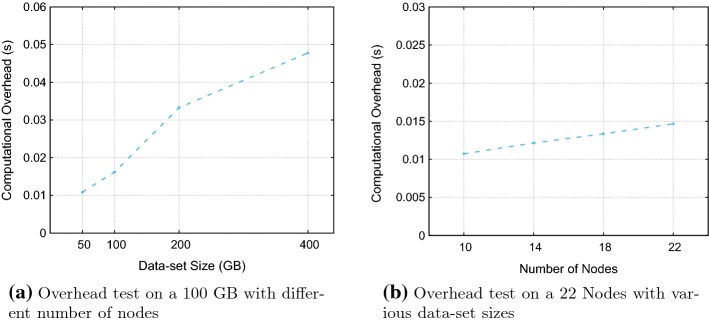
Overhead test

Overhead is another critical aspect of the feasibility of our approach. Therefore, we conducted an experiment to compare the performance gain against the implementation overhead. It is important to note that HaRD does not create an overhead for any other scenarios except the one that replica deletion occurs (i.e., setReplication is triggered with $RF'_i$ is less than $RF_i$). Thus, we only measured the overhead during the replica deletion through. In order to measure the overhead, we injected nanosecond precision time counters at the beginning and the end of our implementation. Then the implementation overhead is calculated by getting difference between time counters. The implementation of HaRD uses WBRD’s code as a base. WBRD already reached insignificant overhead (less than 1.75% of the total time spent in reducing replication). During the development of HaRD, we improved the implementation of WBRD; consequently the efficiency of WBRD increased. Decreasing the replication factor from 10 to 3 consumes 302 s for a 50 GB data set. Addition to Hadoop’s 302 second overhead, HaRD introduces a 10.8 millisecond overhead. Figure 9a, b present the HaRD’s computational overhead for various data-set sizes and number of nodes respectively. In both experiment, we see HaRD’s implementation overheads are significantly less compared to the achieved gain. Moreover, figures show a linear increase in time for the overhead. Thus, it proves that HaRD is highly scalable.

## Conclusion

Current replica management systems adapt the replication factor for ‘hot’ data in order to increase the data locality and achieve better performance, while keeping fewer copies for less frequently accessed data. However, altering the replication factor changes the data distribution. Our previous work identified that replica deletion in Hadoop can be the cause of imbalance in the data distribution and proposed a deletion algorithm for balancing data overall (WBRD). However, WBRD does not consider nodes’ computing capabilities and consequently, leads to sub-optimal performance in heterogeneous clusters. In this paper, we extend the formal definition of the replica deletion problem to heterogeneous clusters. Therefore, we propose a novel cost-effective Heterogeneity-aware Replica Deletion(HaRD) algorithm to use system resources more efficiently. We implemented HaRD on top of HDFS and carried out a comprehensive experimental study to investigate HaRD’s improvements. Experiments show that HaRD improves the system performance by reducing the average execution time by 40% and 8% when compared to HDFS and WBRD. With more concurrent users, the system is fully utilised and the average gains increases up to 60% and 17% compared to HDFS and WBRD, respectively. During tests we observed HaRD’s implementation overhead is significantly less compared to the achieved gain and only 10.8 ms. Moreover, experimental evaluations showed that HaRD’s overhead scales linearly. As future work, we will develop an adaptive replication management framework using the proposed deletion algorithm.

## Data Availability

TPC-H Benchmark: http://www.tpc.org/information/benchmarks.asp. NOAA: https://www.ncdc.noaa.gov/cdo-web/datasets. TestDFSIO and Terasort: https://hadoop.apache.org/.

## Abbreviations

HDFS: Hadoop distributed file system
WBRD: workload-aware balanced replica deletion
HaRD: a heterogeneity-aware replica deletion for HDFS
YARN: Yet another resource negotiator—resource management framework
NN: NameNode
DN: DataNode
*RF*: replication factor
*m*: machine
$PBC_m$: partial block count on a machine
${\mathcal {K}}_m$: a number of containers can be executed simultaneously on a machine
*Cont*: container
*F*: file
*B*: block
TPC: The Transaction Processing Performance Council
NOAA: National Centres for Environmental Information
